# Effects of an empowerment program for survivors of sexual violence on attitudes and beliefs: evidence from the Democratic Republic of Congo

**DOI:** 10.1186/s12939-019-1049-4

**Published:** 2019-09-18

**Authors:** John Quattrochi, Rosalie Biaba, Ragnhild Nordås, Gudrun Østby, Susanne Alldén, Aline Cikara, Esther Namegabe, Christina Amisi

**Affiliations:** 1 Simmons University, Boston, MA USA; 2 International Center for Advanced Research and Training (ICART), Bukavu, Democratic Republic of the Congo; 3 Peace Research Institute Oslo (PRIO), Oslo, Norway; 4 University of Michigan, Ann Arbor, USA

**Keywords:** Empowerment, Sexual and gender based violence, Gender, Attitude, Belief, Democratic Republic of Congo

## Abstract

**Background:**

Women’s empowerment may require women to change their beliefs and views about their rights and capabilities. Empowerment programs often target women who have survived sexual and gender-based violence (SGBV), with the justification that these women may develop disempowered beliefs as a coping mechanism, or face greater barriers to, or derive greater benefits from, the adoption of empowered beliefs and preferences. We investigated an intensive, six-month residential empowerment program (“City of Joy”) for SGBV survivors in eastern Democratic Republic of the Congo (DRC), where more than one in five women have experienced SGBV.

**Methods:**

We asked 175 participants about their beliefs and preferences pertaining to political, financial, and domestic empowerment. Interviews took place immediately before and after participation in the program, and we tested for differences in views of empowerment between entry and exit using paired t-tests and McNemar’s test. We also conducted 50 semi-structured interviews about empowerment with an additional 30 women who had completed the program up to 5 years earlier and then returned to their home community.

**Results:**

Prior to enrolling in the program, participants had fairly empowered views regarding politics, less empowered views regarding finances, and still less empowered views regarding the domestic sphere. After completing the program, participants had significantly more empowered views in all three domains, particularly regarding domestic violence, how families should treat men and women, and women’s economic rights. Participants in their home communities reported taking a more active role in community affairs and speaking out against the mistreatment of women.

**Conclusion:**

This study adds to the evidence that women’s empowerment programs can change participants’ beliefs and thus increase the confidence with which they participate in their communities and support one another.

## Background

The World Health Organization estimates that 35% of women worldwide have experienced either physical and/or sexual intimate partner violence or non-partner sexual violence [1]. In Africa, using data from 18 Demographic and Health Surveys (DHS), [2] found that 29% of women over age 15 had experienced sexual or physical violence in their lifetimes; 22% in the last year. Focusing on the Democratic Republic of Congo (DRC), [3] used the 2007 DHS to estimate that 22% (3.1–3.4 million) of Congolese women had experienced intimate partner sexual violence in their lifetimes, 12% (1.7–1.8 million women) had been raped in their lifetime, and 3% (over 400,000) had been raped in the last year. In another study, focused on eastern DRC, [4] used a population-based survey to estimate that 42% of women in that region had experienced interpersonal violence and 40% had experienced sexual violence (of which 74% was conflict-related). Studies have also documented the widespread use of sexual violence by various armed groups and the Congolese military [5–8]. While the results of these studies are not directly comparable due to differing definitions of violence, it is clear that sexual and gender-based violence (SGBV) affects large numbers of women throughout the world, and Congolese women are no exception.

The consequences of SGBV are dire for survivors and the societies in which they live. SGBV is a violation of survivors’ fundamental human rights, it can severely impair physical and mental health, and it imposes significant costs on their communities [9, 10]. SGBV may result in poor sexual and reproductive health, post-traumatic stress disorder, anxiety, depression or suicide. Communities also often reject or discriminate against women who have been abused. In the eastern DRC this can lead to survivors being perceived as having “lost their value” as women and therefore being shunned in various social settings [11]. As a result of these impairments, survivors, on average, earn lower incomes, produce less, and accumulate less human and social capital compared to women who have never experienced violence [12–14]. Additionally, children who witness violence against their mothers frequently experience psychological trauma that is detrimental to their development, and boys who witness violence are more likely to commit violence [15]. Overcoming or at least addressing the negative consequences of SGBV for female empowerment should therefore be a global priority.

Over the past 30 years, public and private organizations have developed a large number of programs to provide support for survivors of SGBV. This study focuses on Panzi Foundation’s City of Joy (CoJ), a transformational leadership community for women survivors of violence, located in Bukavu, South Kivu Province, the Democratic Republic of Congo (DRC). Conceived, owned, and run by local Congolese, the CoJ first opened its doors in June 2011. The 6-month residential program is designed to heal women “from their past trauma through therapy and life skills programming while providing them with the essential ingredients needed to move forward in life: love and community.”^1^ Participants are recruited in collaboration with rural associations affiliated with Panzi Hospital. To be eligible, women must be 15 to 31 years old, and survivors of SGBV; they must have already received medical care, if necessary; and they must agree to live in the CoJ compound for 6 months without their partners and children (if any). CoJ particularly encourages women with leadership potential to participate. Participants must also sign an act of commitment to comply with the standards established by CoJ through the 10 guiding principles^2^ or channels offered to “turn pain into power” for the participants.

About 30 h of classes and training are provided each week during the 6 month program. Roughly three-quarters of the formal CoJ program consists of psychotherapy (approximately 22 h/week), which includes individual and group therapy, as well as meditation. The rest of the program consists of the following (not all courses run for the full 6 months): literacy (5–7 h/week), gender rights (6 h/week), theatre (5 h/week), civic and political education (5 day seminar), self-defense (6 sessions, 2 h/week), social communication (6 h/week), entrepreneurship (3 day seminar), computer literacy (6 h/week), judicial competence, organization and proceedings, and children’s rights (1 day seminar), peaceful coexistence and conflict resolution (1 day seminar), comprehensive sexuality education (2 h/week), animal husbandry, agriculture and ecology (3 h/week), artistic sewing (6 h/week), culinary arts (6 day seminar), soap making (5 day seminar), knitting/crocheting (4 h/week), nutrition (2 h/week), and physical education (optional).

In this paper, we set out to answer three related research questions. First, before participating in the CoJ program, what views do participants hold about women’s political, financial, and domestic empowerment? Second, have these views changed by the end of the program and, if so, how? Third, how is life different, if at all, for participants after they have returned home, compared to life before the CoJ program, and do the participants attribute the differences to changes in their own views?

## Methods

### Study setting

The CoJ compound is located in the Panzi neighborhood of Bukavu, the capital of South Kivu Province, a city of approximately 1 million inhabitants on the southern shore of Lake Kivu, in the eastern region of the Democratic Republic of Congo. There has been a series of humanitarian crises in the region for over 20 years, largely as a result of on-going armed conflict and low state capacity. The influx of refugees from Rwanda in 1994 coupled with long-standing tensions relating to land rights, citizenship, and ethnicity, led to the First (1996–7) and Second (1998–2003) Congolese Wars. The Second Congolese War directly involved eight African nations and 25 armed groups, and was the deadliest war in modern African history [16]. Despite the formal end to the war in July 2003, dozens of armed groups have continued to operate in South Kivu and elsewhere in eastern Congo over the subsequent 15 years. Many of these armed groups commit sexual violence, and the prevalence of civilian-perpetrated rape is also high.

### Entry and exit survey

We administered a survey to all women who entered the CoJ program (175 in total) in June 2015 and January 2016, using a closed-form questionnaire with a maximum of 168 questions (there could be fewer due to skip patterns). We also administered another survey of similar length to these same women when they graduated from the program 6 months later. The entry and exit surveys contained identical questions about the participants’ views on political, financial, and domestic empowerment. The entry survey also included questions about basic demographic information, and the exit survey contained questions about satisfaction with the CoJ program and perceptions of the future.

We recruited local women in Bukavu with university degrees to serve as enumerators. Ten enumerators were selected and trained to carry out the surveys on electronic tablets. The enumerators conducted the interviews in a private setting, assured the interviewees that participation was voluntary and that their responses were confidential. The enumerators also explained that they were part of an independent research team, unaffiliated with the CoJ program. The questionnaire was written in French and English and then translated into Swahili, and programmed in the tablets using Open Data Kit (opendatakit.org). The surveys were conducted in Swahili, with French or Mashi (a local language) used as needed. The enumerators recorded participants’ answers in the tablets, as some women interviewed were not literate and would have had difficulties filling out the survey themselves.

### Statistical analysis

We tested for differences in views of empowerment between entry and exit. For Likert-scale questions we used paired t-tests, which have been shown to have higher power and lower Type I error than Mann-Whitney-Wilcoxon tests for data distributions similar to those we found [17]. For questions with binary answers, we use McNemar’s test. Because we are testing multiple hypotheses, we also use the [18] method to control the false detection rate [19].

### Qualitative interviews and focus group discussions

We conducted six focus group discussions (FGDs) with 60 randomly selected CoJ participants (10 per group) from the cohort that began in January 2015. They were 2–4 months into the six-month program at the time of the FGDs. These interviews were used to develop the questionnaire used for the entry and exit surveys for the next cohorts.

We developed an interview guide and held a basic training on interview technique (focusing on semi-structured interviews) in September 2015. We conducted 50 semi-structured interviews with 30 women who had returned to their home communities after graduating from CoJ between 2011 and 2015. To ensure the safety of our research team, we interviewed women who lived in relatively secure areas within an eight-hour drive of Bukavu and who had graduated from CoJ at least 4 months earlier. The software MaxQDA was used to code and analyze all of the qualitative data.

## Results

For the quantitative survey, we interviewed 89 women as they entered the program in June 2015. Three women were deemed ineligible for the CoJ program (after our baseline survey) due to being pregnant, and were replaced by other women within 1 month. One woman also arrived after our baseline survey. In January 2016, we interviewed 90 women as they entered the program. One woman was deemed ineligible due to pregnancy and was replaced, again within 1 month. We interviewed all of the women who completed the program. Thus we interviewed a total of 175 women both before and after participation in the program (86 in 2015 and 89 in 2016), and we interviewed five women only after participation in the program. We restricted our analyses to those women who were interviewed twice (*n* = 175).

Compared to the broader population of Congolese women, CoJ participants were younger, less likely to be married, and better educated; which reflects the selection process of CoJ. Participants were between 15 and 31 years old, with 63% being 18–20 years old (Table 1). Most resided in South Kivu province (72%), with another 20% from North Kivu. The majority were single (81.7%), with 5.1% married and 16.9% separated. In the broader population, 46.5% of Congolese women aged 15–49 are married [20]. Only 6.9% of participants had no education, compared to 15.4% of Congolese women aged 15–49. 59.9% of participants had at least some secondary education, compared to 44% of Congolese women aged 15–49. 74.9% of participants owned a mobile phone before participating, and 56.6% came from a home with a straw roof. Just over half identified as Protestants (54.3%), with most of the rest identifying as Catholic (36.6%); among all Congolese women aged 15–49, 29.7% identify as Catholic and 26.8% identify as Protestant. Participants were members of over 12 different ethnic groups, with Shi being the most prominent (24.0%); there are approximately 400 ethnic groups in Congo overall [20].

**Table 1:** Characteristics of City of Joy participants

Variables	
Age, Mean (Min, Max)	20.4 (15, 31)
Births, No. (%)	
*0*	86 (49.1%)
*1*	70 (40%)
*2*	14 (8%)
*3*	4 (2.3%)
*4*	1 (0.6%)
Marital Status, No. (%)	
*Married*	9 (5.1%)
*Single*	143 (81.7%)
*Separated*	23 (13.1%)
Level of Education, No. (%)	
*None*	12 (6.9%)
*Some primary*	41 (23.4%)
*Completed primary*	17 (9.7%)
*Some secondary*	86 (49.1%)
*Completed secondary*	17 (9.7%)
*Some tertiary*	2 (1.1%)
Own mobile phone, No. (%)	131 (75%)
Home has straw roof, No. (%)	99 (56.6%)
Religion, No. (%)	
*Catholic*	64 (36.6%)
*Protestant*	95 (54.3%)
*Muslim*	3 (1.7%)
*Other*	13 (7.4%)
Province of residence, No. (%)	
*South Kivu*	126 (72.0%)
*North Kivu*	35 (20.0%)
*Maniema*	12 (6.9%)
*Other*	2 (1.1%)
Tribe, No. (%)	
*Shi*	42 (24.0%)
*Bembe*	11 (6.3%)
*Barega*	14 (8.0%)
*Havu*	24 (13.7%)
*Nande*	9 (5.1%)
*Fuleru*	17 (9.7%)
*Tembo*	8 (4.6%)
*Kinyarwanda*	5 (2.9%)
*Nyabwishi*	4 (2.3%)
*Nyindu*	1 (0.6%)
*Kikusu*	2 (1.1%)
*Hunde*	22 (12.6%)
*Other*	16 (9.2%)

### Participants’ views on political empowerment

**Fig. 1:**
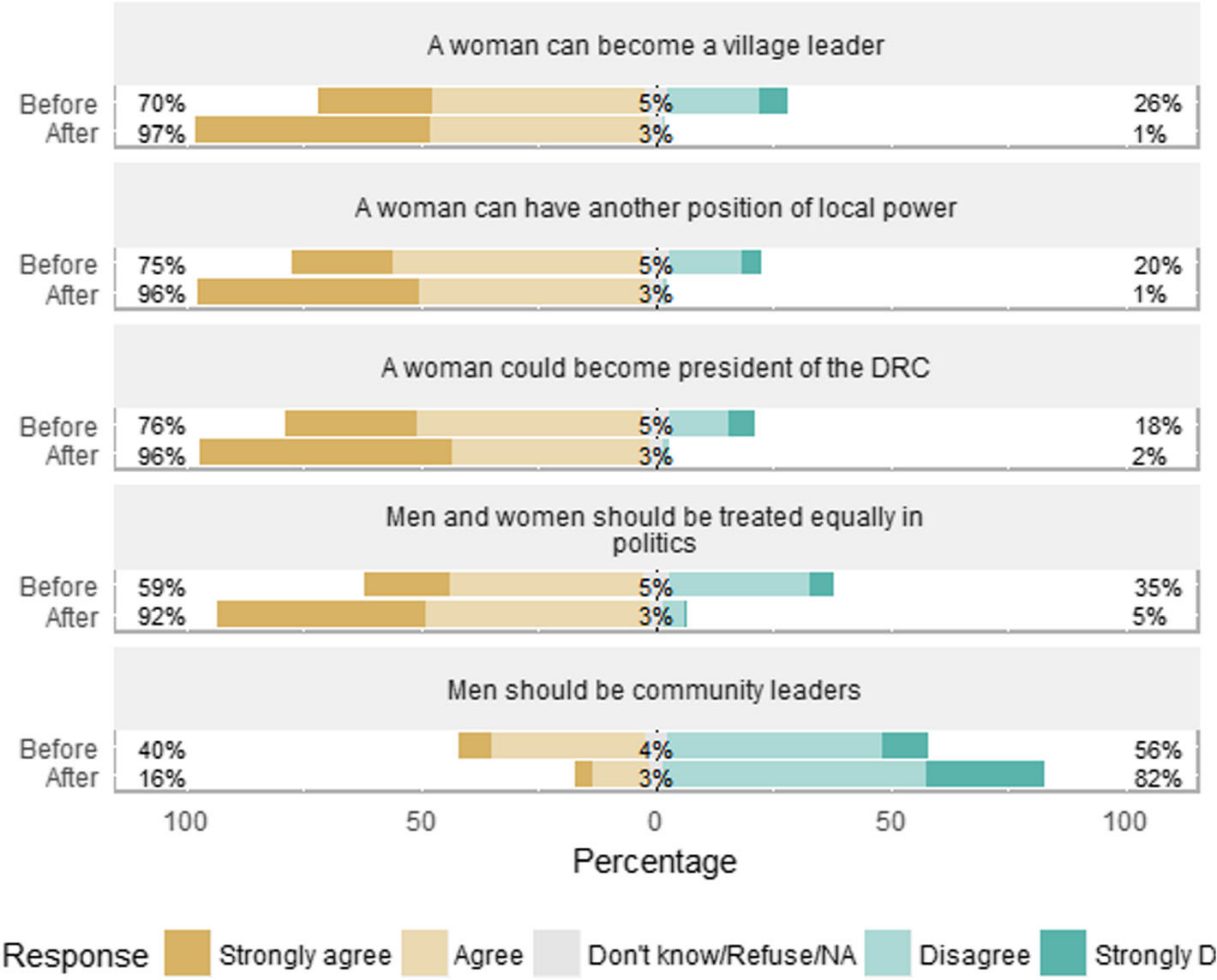
Participants’ views on political empowerment, before and after the program. Percent of participants in the City of Joy empowerment program (*n* = 175) agreeing or disagreeing with the statements in the figure, before beginning the six-month program (“Before”) and after completion (“After”)

**Fig. 2:**
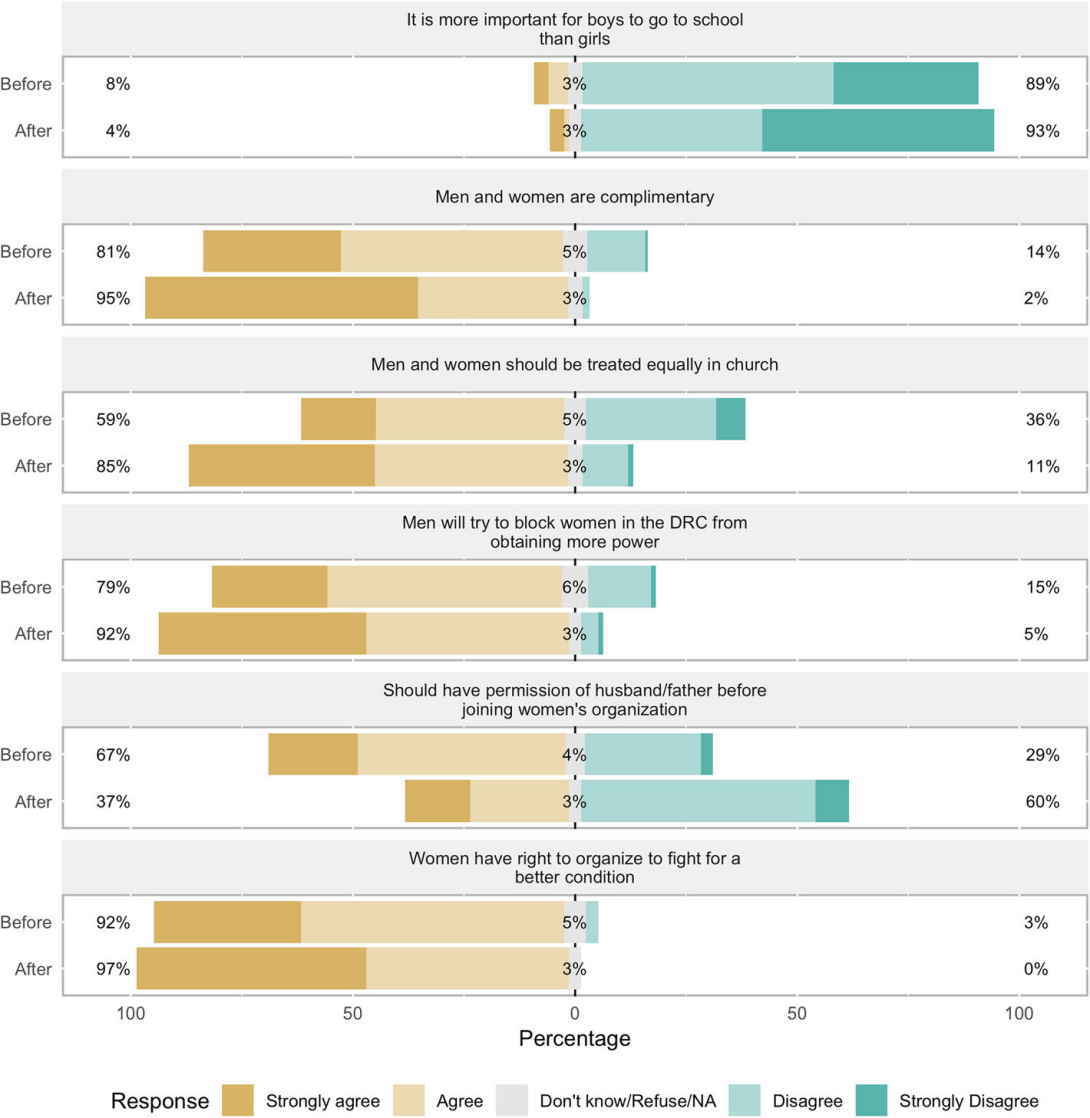
Participants’ views on political empowerment, before and after the program. Percent of participants in the City of Joy empowerment program (*n* = 175) agreeing or disagreeing with the statements in the figure, before beginning the six-month program (“Before”) and after completion (“After”)

Participants had fairly empowered views about politics before the CoJ program (Table 2; Figs. 1 and 2). Over 90% believed that women have the right to organize to fight for better conditions in the DRC, and nearly 90% agreed that a woman can be a leader and did not agree that it is more important for boys to go to school than girls. About 80% agreed that men will try to block women in the DRC from obtaining more power, and that men and women are “complimentary”. 70% or more agreed that a woman could become a village leader, have another position of local power, or become president of the DRC, and over 70% felt comfortable speaking up in church, mosque, or in community meetings. 59% agreed that women and men should be treated equally in politics, 59% agreed that women and men should be treated equally in church, 60% said they are comfortable expressing a disagreement with a religious leader, and 56% said they are comfortable expressing a disagreement with a community leader. 67% agreed that a woman should have her husband or father’s permission before joining a women’s organization. Forty percent agreed that men should be community leaders, and only 21% were aware of the seven UN resolutions on women’s rights.^3^

**Table 2:** Participants’ views on political empowerment before and after the City of Joy program

Question	Response	Before	After	Difference	*p*-value
Do you think that a woman can be a leader?	Yes	89%	99%	10%	< 0.01
Are you aware of the existence of sevenresolutions on women’s rights?	Yes	21%	55%	34%	< 0.01
Do you feel comfortable speaking up or discussing your opinions in the following settings:					
Church/Mosque	Yes	75%	98%	23%	< 0.01
Community meetings	Yes	77%	97%	20%	< 0.01
Do you feel comfortable expressing your opinion if you disagree with the following people:					
Community leader	Yes	56%	96%	40%	< 0.01
Religious leader	Yes	60%	98%	38%	< 0.01

Notes: *P*-values were calculated using McNemar’s test under the null hypothesis of no change in the answer between before and after. *P*-values were adjusted for multiple hypothesis testing using [18]

After completing the CoJ program, over 80% of the participants gave answers that we judge to be the most empowered views on the issues listed in the previous paragraph, with only two exceptions (Table 2 and Figs. 1 and 2): 37% still agreed that a woman should have a father or husband’s permission before joining a women’s organization (meaning 63% did not deem such a permission to be required), and only 55% were aware of the seven UN resolutions on women’s rights, which the participants were informed about during the program.

All of the differences in participants’ views before and after the program are statistically significant at the 0.05 level, even after adjusting for multiple hypothesis testing. This holds across all questions in all three domains of empowerment: political, financial, and domestic. (See Additional File 1 for details on the Likert scale variables depicted in the figures).

### Participants’ views on financial empowerment

**Fig. 3:**
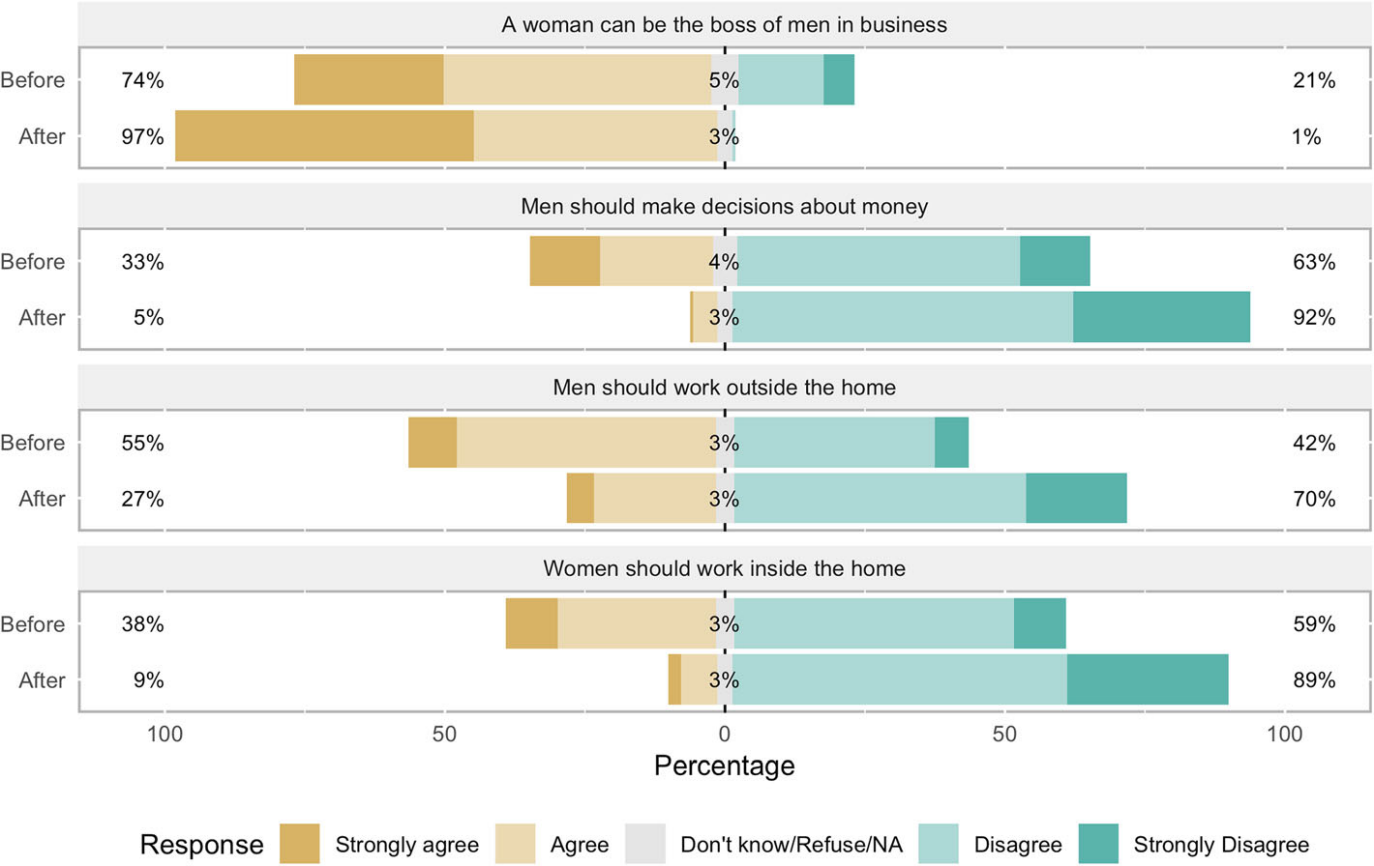
Participants’ views on financial empowerment, before and after the program. Percent of participants in the City of Joy empowerment program (*n* = 175) agreeing or disagreeing with the statements in the figure, before beginning the six-month program (“Before”) and after completion (“After”)

**Fig. 4:**
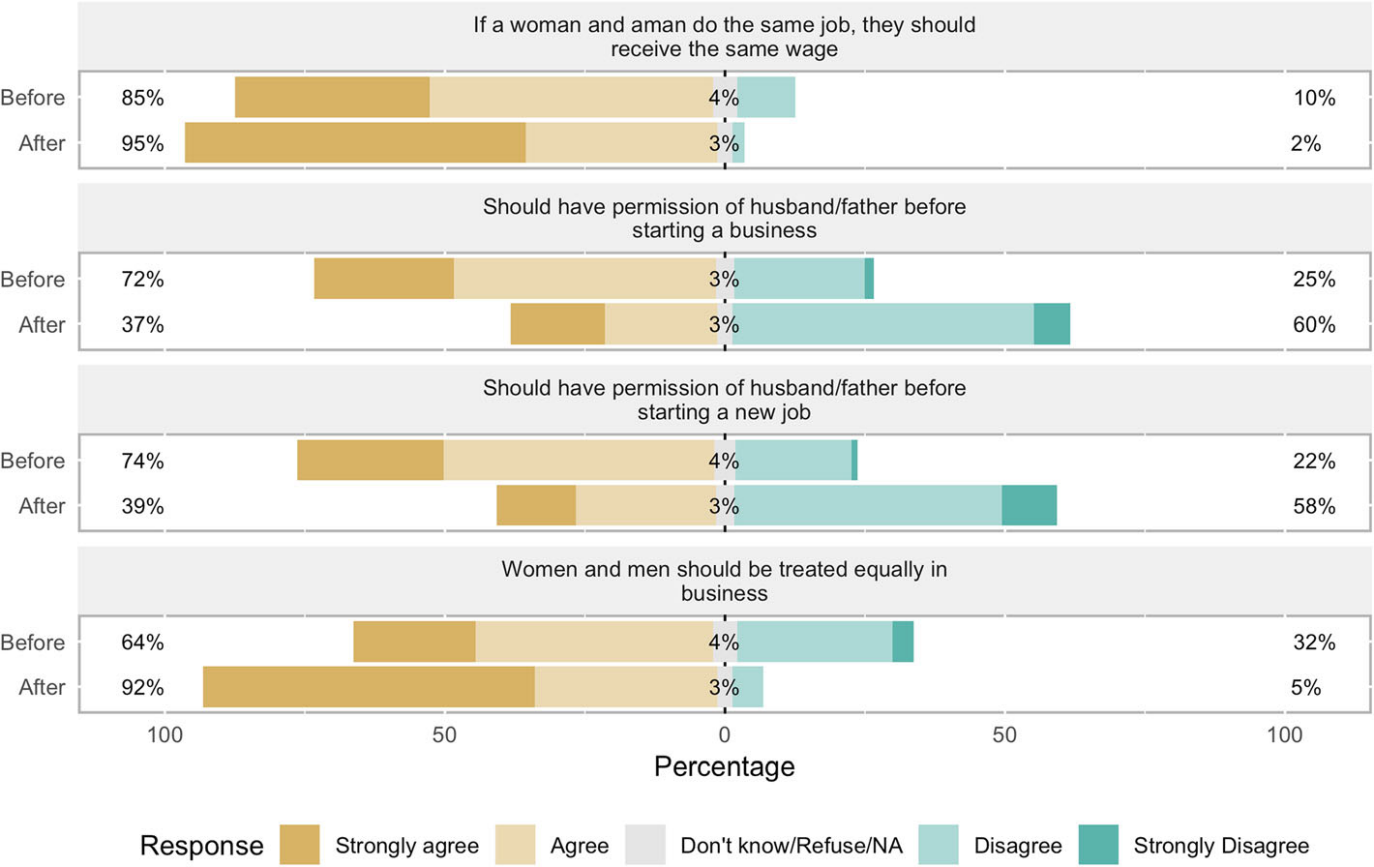
Participants’ views on financial empowerment, before and after the program. Percent of participants in the City of Joy empowerment program (*n* = 175) agreeing or disagreeing with the statements in the figure, before beginning the six-month program (“Before”) and after completion (“After”)

Before participating in the CoJ program, participants held a mix of views regarding financial empowerment (See Table 3 and Figs. 3 and 4). 85% of participants agreed that if a woman and a man do the same job, they should receive the same wage. 77% agreed that a woman has the right to inherit land from her father, husband, or next of kin, and of those who agreed to the statement, 91% said they would fight for that right. 74% agreed that a woman can be the boss of men in business. 64% agreed that women and men should be treated equally in business. 55% agreed that men should work outside the home and 38% agreed that women should work inside the home. 33% reported that only men make decisions about money in the household; 59% said that decisions about how to use money were made by themselves or joint with someone else.

**Table 3:** Participants’ views on financial empowerment before and after the City of Joy program

Question	Response	Before	After	Difference	*p*-value
Does a woman have the right to inherit land from her father/husband/next of kin?	Yes	77%	96%	20%	< 0.01
If yes, is this a right you would fight for?	Yes	91%	100%	9%	< 0.01
If you earn some money, do you have to ask for someone’s permission before you spend it?	No	44%	70%	26%	< 0.01
Who decides how to use money?	“Me” or “Joint decision with someone else”	59%	82%	23%	< 0.01

Notes: *P*-values were calculated using McNemar’s test under the null hypothesis of no change in the answer between before and after. *P*-values were adjusted for multiple hypothesis testing using [18]

Before the program, many participants felt the need to have permission from their husband or father before making financial decisions. 72% agreed that they should have permission before starting a business, and 74% before starting a new job. Only 44% said they did not need someone’s permission to spend money they had earned.

After completing the CoJ program, participants had markedly more empowered views about financial matters. Over 90% of women agreed that a woman can be the boss of men (up from 74%), that women and men should receive the same wage for the same job, that women and men should be treated the same in business, and that a woman has the right to inherit land. 92% did *not* agree that men should make decisions about money in the household. Only 37 and 39% agreed that they should have permission from their husband or father before starting a business or a new job, respectively. 70% said they do not have to ask for permission before spending money they’d earned. 82% said that decisions about money were made by themselves or jointly. Finally, 70 and 89% did not agree that men should work outside the home and women should work inside the home, respectively.

### Participants’ views on domestic empowerment

**Fig. 5:**
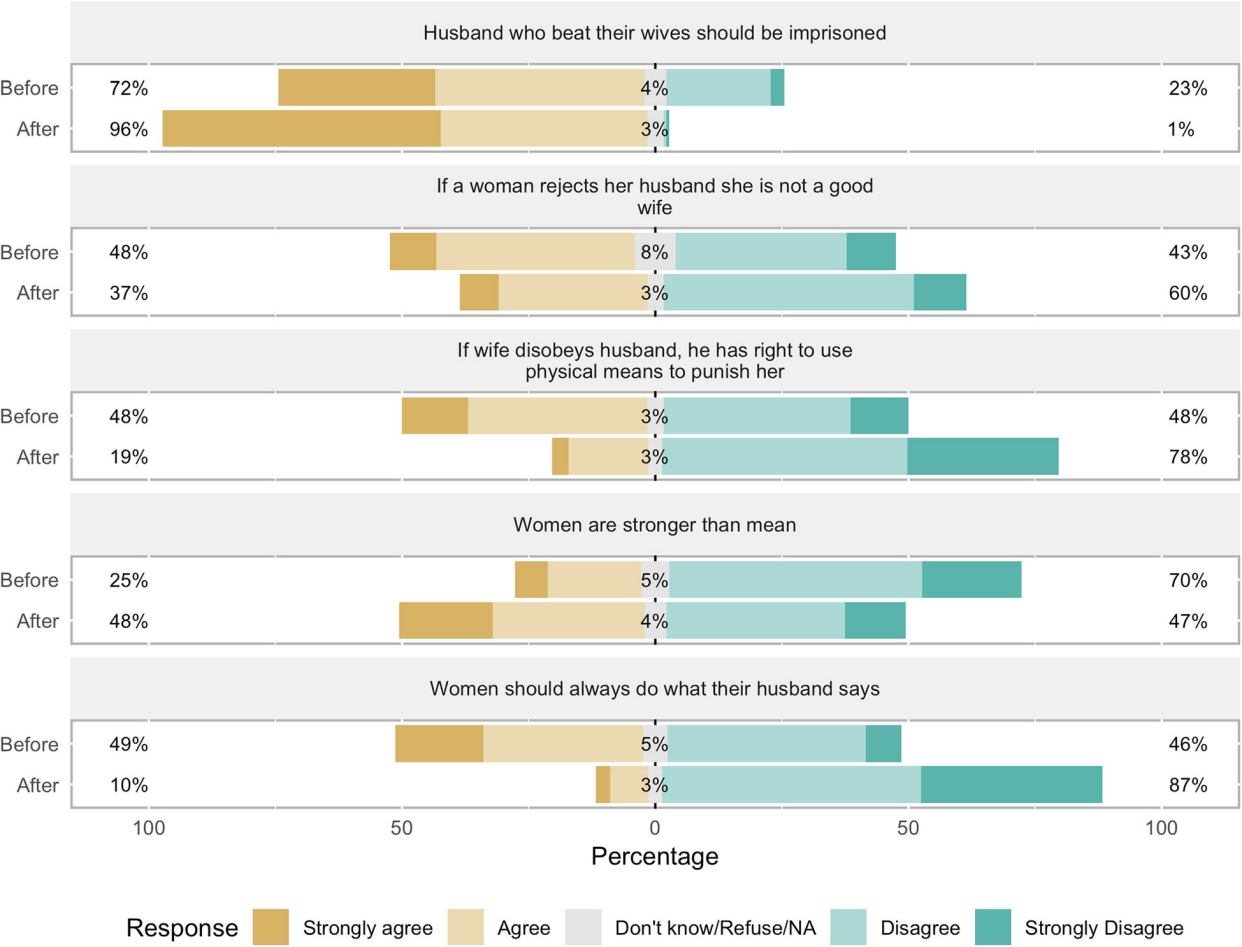
Participants’ views on domestic empowerment, before and after the program. Percent of participants in the City of Joy empowerment program (*n* = 175) agreeing or disagreeing with the statements in the figure, before beginning the six-month program (“Before”) and after completion (“After”)

**Fig. 6:**
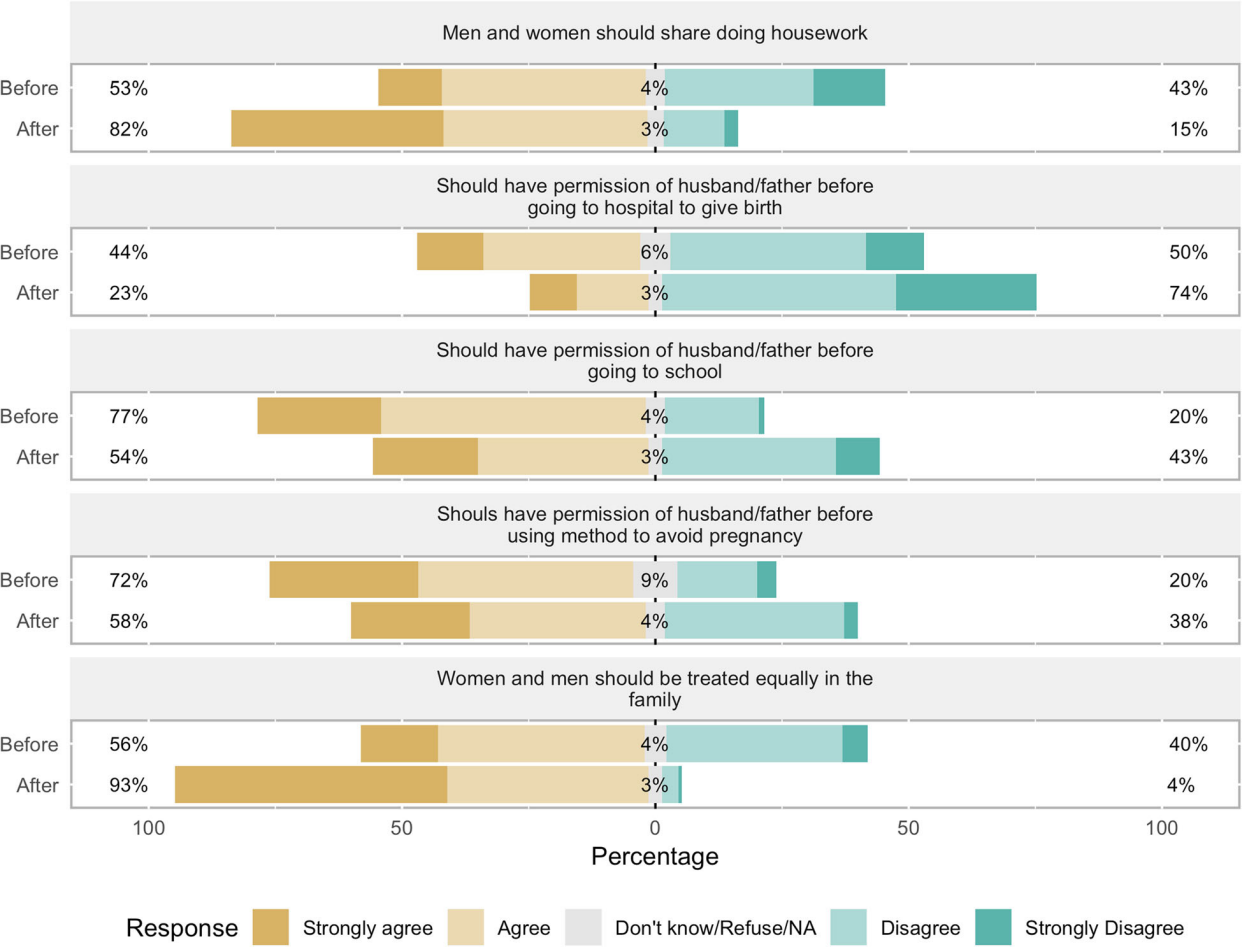
Participants’ views on domestic empowerment, before and after the program. Percent of participants in the City of Joy empowerment program (*n* = 175) agreeing or disagreeing with the statements in the figure, before beginning the six-month program (“Before”) and after completion (“After”)

Before the CoJ program, participants held rather unempowered views on domestic issues, although most were comfortable expressing disagreement with husbands (83%), fathers (77%), mothers (90%), brothers (87%), and sisters (89%), and 77% were comfortable speaking up at family gatherings (See Table 4 and Figs. 5 and 6). 72% agreed that husbands who beat their wives should be imprisoned. However, 48% agreed that if a wife disobeys her husband, he has the right to use physical means to punish her. 49% agreed that a woman should always do what her husband says, 40% agreed that a woman should be ready to have sex with her husband at any time, 48% agreed that a woman who rejects her husband is not a good wife, 53% agreed that men and women should share doing housework. Only 25% agreed that women are stronger than men.

**Table 4:** Participants’ views on domestic empowerment before and after the City of Joy program

Question	Response	Before	After	Difference	p-value
Do you feel comfortable speaking up or discussing your opinions in family gatherings?	Yes	77%	95%	18%	< 0.01
Do you feel comfortable expressing your opinion if you disagree with the following people:					
Husband	Yes	83%	97%	14%	< 0.01
Father	Yes	77%	93%	17%	< 0.01
Mother	Yes	90%	97%	8%	< 0.01
Brother	Yes	87%	95%	8%	0.023
Sister	Yes	89%	97%	8%	< 0.01
Do you believe a woman should be ready to have sex with her husband/partner at any time?	No	60%	88%	28%	< 0.01
Who decides in your home for you to have access to family planning?	“Woman alone” or “Husband and wife”	41%	79%	38%	< 0.01
In your household, who should eat first?	“All together”	31%	41%	10%	0.034
Who should decide when you should get married?	Me	64%	88%	24%	< 0.01

Notes: *P*-values were calculated using McNemar’s test under the null hypothesis of no change in the answer between before and after. *P*-values were adjusted for multiple hypothesis testing using [18]

Most participants agreed that that a woman should have her husband or father’s permission before key domestic decisions: 77% for decisions about going to school; 72% for using a method to avoid pregnancy; 44% before going to the hospital to give birth. Only 31% said that the decision to have access to family planning was the woman’s alone or that of the husband and wife together. 64% said that they (rather than family members or others) are the person who should decide when they marry.

After completing the CoJ program, participants’ views had become more empowered on all questions. The largest shifts were seen in response to questions on who decides about family planning (38 percentage point increase toward “woman alone” or “husband and woman together”), whether a woman should always do what her husband says (39 percentage point increase toward “disagree/strongly disagree”), and whether men and women should be treated equally in the family (36 percentage point increase toward “agree/strongly agree”). Many more women also reported not believing that a husband has the right to use physical means to punish his wife (30 percentage point increase), not believing that a woman should be ready to have sex with her partner at any time (28 percentage point increase), and believing that they alone should decide when to marry (24 percentage point increase).

### Longer-term impacts

The women who participated in semi-structured interviews and focus group discussions had graduated from CoJ 4–48 months earlier and then returned to their home communities in various parts of South and North Kivu provinces. These women talked about positive developments due to what they had learned about their rights and due to their new capacities and skills. Overall, they reported feeling more confident, independent, and able to speak out compared to before the CoJ program.

Many of the women interviewed talked about the importance of the trainings they received at CoJ and how it had helped them upon their return back home. Some talked about their role as trainers and how the message they had learned could be transferred to other women. This also requires that they be more aware of the suffering of other women in their communities. “When I learnt these lessons from CoJ and returned home, I figured out that women are really suffering in our village.”^4^ One woman explained that when she returned home after CoJ she was asked if she had learned any skills. She responded that she had, but more importantly, “I have learned to understand how women are mistreated and how we should live”,^5^ and the importance of teaching others about this as well. “We women, we can contribute to change because after we have received training we will train others.”^6^ Reflecting upon the trainings received at CoJ, she concluded that, most importantly, she had learned “to know how to live” and how to protect herself against harm and those who want to hurt women. Another woman stated, “I see City [of Joy] as a lamp that was switched on to teach others. Teaching girls about strategies can allow them to leave a disastrous situation, for a better situation. We also teach men […]; that is how things can change.”^7^

Women reported changed perceptions of gender roles and of women’s abilities, and that this gives them the power to act for change. Although their fight for women’s rights is not well coordinated, some of the women from the CoJ program stand up and advocate for the rights of their fellow women, especially when they are ill-treated and their rights are being openly violated. For example, one woman interviewed explained her reaction when she saw a man mistreating his wife, as well as how this has changed how the villagers see her:

“A man was beating his wife, I came forth and said you have no right to do that, and the woman has the right to live in freedom. He wanted to chase his wife and I told him that his wife shall not go anywhere because they have already had children, they have a piece of land that they bought together and built. They used to rent a house before, so this was not the time to chase her. He also makes mistakes, why not his wife? She is not a cow to be beaten. I intervened a lot to prove that this was not life. If not, both should leave the house and let the house belong to the children. The man asked me where I have learnt all of these things. I told him that these are things he is supposed to know regardless of where I learnt them. That is why in our community men say that I have many things in my mind and that people should be careful with me.” 8

The last part of the quote reveals that this woman is now regarded as dangerous; her new knowledge makes her a perceived threat to some members of the community. This highlights the tension that can arise when women challenge the social norms in their communities.

Other participants talked about the importance of the trainings to increase their own self confidence and to understand that they have value.^9^ Accepting one’s weaknesses and turning them into strengths was something that many women highlighted as key for their own personal development during their stay at CoJ.^10^ One woman explained that since she started to believe more in herself, she also felt better both physically and psychologically, and it allowed her to sleep and eat better.^11^ “My worries have disappeared”, stated another woman, “because I have accepted myself.”^12^ Knowing that other women had also suffered helped some women overcome their own trauma.^13^

Skills-training such as agro-pastoral techniques has helped women improve their productivity, which in turn has increased their revenues.^14^ Increased revenues result in increased independence. “We need to look for our own money so we do not stay dependent on others.”^15^ One woman stated that “since I have left City of Joy, I do not wait to depend on anyone, but I have started to work for myself and earn my own money.”^16^ Another woman explained that she felt a change since leaving CoJ because she had been taught that everyone needed to work: “Before CoJ I thought it was only my father and my mother who needed to work to look after me, but since I left [CoJ] I know that a person can work to become independent and have a good life.”^17^

A CoJ graduate from Nyangezi gave an example from her life when she went looking for work at a construction site:

“I went to ask for work, I saw an engineer there and I told him that I was looking for work. There were only men working there […]. I asked if only men merited working. I did not get a job that day, but later on they were recruiting and since then I have a job there.”^18^

Women also testified about increased respect from others after their return from CoJ. “I was the reason for their rejection because I showed them my fear and shame […] I stayed at home in tears,” which is no longer the case, explained one graduate.^19^ “They know I am a leader,” said another.^20^ “[The community] has started to respect me, even in providing advice and speaking to them. Before they said ‘who is she to speak up?’, but now they have started saying, ‘she is saying something important.”^21^

Many women expressed an increased confidence in speaking up in public. One woman explained that before going to CoJ she was afraid of speaking up, thinking that others would criticize her for not knowing anything, but after her stay at CoJ she “has woken up.”^22^ “I am a person and I can speak up in front of people; I have arrived to where I was not capable of going before”, stated one young graduate.^23^ “When I left City of Joy I had the idea that when I return home I will not accept that anyone walks over me, and I will not accept seeing any other woman being walked over,”^24^ expressed one woman. She went on to explain that “if I learn that a woman like me is being dominated, I will not accept it and I will go there and say something […] they can do whatever they want to me, hit me or not, but the important thing for me is to speak up”. This lack of fear was reiterated by another woman interviewed who explained that before she was at City of Joy she was afraid, but today “even if someone asks me to speak in front of the President I will do it […] Even if I die, I will die having spoken up”.^25^

In terms of female leadership, the graduates talked about several local female politicians and how they believed them capable of running for office. They highlighted these women’s capability to understand the suffering of women,^26^ and how they would want to promote other women^27^ as important strengths. At least one of the women we interviewed 3 months after graduation had a strong ambition to become head of their village and to seek such power to fight for the rights of the women in particular. Several women had joined women’s organizations in their communities.

Unsurprisingly, given the difficulties facing women in eastern DR Congo, there are some issues in their communities that will take time to change. For example, as expressed by a CoJ graduate from Kamanyola: “Women’s rights are not respected in our village, women are terribly humiliated, to the extent that their husbands consider them as objects, I am not sure what to say, especially when the husband is drunk and he beats his wife”.^28^ Although the women feel empowered themselves, they cannot be solely responsible for changing a patriarchal society where violence has become an accepted way of solving disagreements.

## Discussion

Scholars and policymakers are increasingly interested in women’s empowerment, as it has been linked to individual well-being and societal prosperity [21]. Programs and policies to promote empowerment have proliferated, and yet no consensus exists about what works and why. In this study, we compared young female SGBV survivors’ views and beliefs about political, financial, and domestic gender roles before and after they participated in a unique residential empowerment program (CoJ) that included leadership training and a significant therapeutic component. To investigate how the CoJ program influenced the participants’ relevant attitudes and beliefs regarding female empowerment, we asked three research questions: (1) What views do participants hold about political, financial, and domestic empowerment of women before joining the empowerment training? (2) How did these views change by the end of the program, if at all? (3) How is life different, if at all, for participants after they have returned home, and do participants attribute changes to their own more empowered views?

We show that, prior to the program, the participants in the program did have quite empowered views on some of the dimensions we were interested in. For example, most participants agreed that women could hold positions of public authority. But at the same time a significant minority were not comfortable expressing disagreement with community and religious leaders. Over half reported that a woman should have the permission of her father or husband prior to joining a women’s organization. Regarding financial empowerment, most participants held empowered views prior to the program, but many women: disagreed that a woman can be the boss of men in a business; reported that men make decisions about money; agreed that women should work inside the home; and reported that they needed to ask for someone’s permission before spending money.

In regards to domestic empowerment, prior to participation, half of the women agreed that a husband has the right to use physical means to punish a wife who disobeys him; almost half agreed that a wife should be ready to have sex with her husband at any time; and over half agreed that a woman should have her husband or father’s permission before going to school or using contraception.

The relatively high share of women that expressed empowered views prior to joining CoJ might be somewhat surprising. We believe this might be attributed to the selection process of the empowerment program. The selection is in part focused on identifying young women in particularly vulnerable positions who need the assistance the program can provide. On the other hand, the program also makes an assessment of who is likely to benefit; in other words, who appears to have the potential to “transform pain into power”, which is the motto of the program. In that selection process, those who are selected might be already showing signs that they are open to thinking in new ways about their role in society. Currently, there is no representative survey to compare the participants’ views to those of other young women in the DRC, and this could influence the external validity of the study. Nonetheless, the results we provide are in themselves robust and interesting from the perspective of validating and improving similar programming in the DRC and comparable contexts.

After completion of the CoJ program, participants’ views had become more empowered across all three dimensions. For example, over 95% reported feeling comfortable expressing their opinions, even in disagreement, in community settings. Ninety-eight percent agreed that a woman has the right to inherit land and reported that they would fight for that right. The percent agreeing that it is their own decision to marry and that a woman needn’t be ready to have sex with her husband/partner at any time increased by over 24 and 29 percentage points, respectively. The percent of women aware of the seven UN resolutions on women’s rights increased by 34 percentage points, but at baseline only 22% of women were aware. This suggests that there is much more to be done to translate these resolutions in ways that are meaningful for Congolese women.

Despite the large increases in empowered views, there remained significant minorities of women with unempowered views after the program. For example, 30% report having to ask someone’s permission to spend money that they earned; 18% reported not being involved in decisions about how to use money; 12% report that a woman should be ready, at any time, to have sexual relations with their partner; and 21% report that they are not involved in decisions about access to family planning. While it is unlikely that any empowerment program can change the views of all participants, we see room for improvement in the CoJ program. This could take the form of modified program activities, increased duration or intensity, or activities targeted at participants whose views seem most resistant to change.

In-depth interviews of a subset of participants 4–48 months after program completion revealed that participants are emboldened to take on more active roles in their community and to share what they have learned with others. They are more likely to speak out against what they judge as wrong, and to seek public positions that would allow them to make broader changes. These findings support the philosophy of CoJ that young women who have grown up in societies where women’s rights are often violated can nonetheless be trained to become change agents.

We did not interview other community members to investigate how they feel about survivors of sexual violence or women who have graduated from an empowerment program. Recent studies have found very negative community perspectives towards survivors of sexual violence, and that their communities perceive that they have “lost their value”, particularly in the social sphere [11], making reintegration and empowerment of survivors particularly important but also a demanding process [22].^29^ It is likely that social norms, particularly around masculinity and gender roles, will need to change before survivors and empowered women can be fully reintegrated into their communities.

That the CoJ program changed women’s attitudes and beliefs is perhaps not surprising given the duration and intensity of the program. The rapidly growing literature on female empowerment includes several examples of less intensive interventions that were shown to change participants’ attitudes and beliefs. Importantly, several of these studies also link the changes in attitudes and beliefs to changes in behaviors, which suggests that the same may be true for CoJ graduates. Participants in other programs reported fewer incidents of intimate partner violence [23], less sexual assault [24, 25], and higher age at first pregnancy [24].

There are several limitations to this study. It was not feasible to include a control group, therefore we do not have a credible counterfactual for how the views of the CoJ participants might have changed if they had not, in fact, participated. However, given the relatively short duration of the program, and the dramatic changes in participants’ views on empowerment, we are confident that the observed changes were in fact due to the program. This is strengthened by the fact that we interviewed participants in several different cohorts, ensuring that our results were not driven by factors cotemporaneous with the program (e.g. a media campaign promoting women’s empowerment). Also, we cannot conclude how a program like this might have changed participants’ attitudes and beliefs if the selection criteria were different (for example, if the participants were older and/or admission was not based on perceived leadership potential).

As with any study using self-reported measures, social desirability bias is a concern. The process of contacting former CoJ participants may select for people who are more likely to have benefited from the program, or it may signal to them that we only want to hear positive feedback. However, our interviewers, who had similar backgrounds to the respondents, were trained to establish trust and rapport with the goal of eliciting honest responses.

Another limitation is that there is no scientific consensus on how best to measure empowerment. One can argue about the appropriateness of the questions that we’ve included here. We chose our questions based on focus groups and discussions with eastern Congolese women about what was most relevant to them.

When considering the generalizability of these findings, we should keep in mind that participants were not randomly selected from the female population of eastern DRC. Rather, they are survivors of SGBV who were identified by local NGOs as young women with leadership potential. That said, it is likely that there are many women with comparable potential, both in the DRC and elsewhere, who would similarly benefit from a program like CoJ.

## Conclusion

This study provides evidence that the CoJ empowerment program changes participants’ attitudes and beliefs in a positive way. Women who have returned to their communities after the program see themselves as more confident and independent, they speak out more, and they support other women in their communities. Even in contexts as challenging as eastern DRC, where many traditional cultures see women as subservient to men, and where sexual violence has been weaponized over 20 years of armed conflict, it is possible to create a community that fosters women’s empowerment. Further research is needed to investigate the possibility of scaling up the CoJ model, to measure the longer-term influence of graduates in their home communities, and to assess whether social norms around gender-based violence and women’s role in Congolese society are changing.

## Supplementary information

**Additional file 1: Table S1.** Statistical tests for differences in empowerment on Likert scale before and after City of Joy.

## Data Availability

The datasets generated and/or analyzed during the current study are not publicly available due to concerns about participants’ privacy but are available from the corresponding author on reasonable request.

## Footnotes

1
https://cityofjoycongo.org/

2 These principles are: (1) Tell the truth (2) Stop waiting to be rescued; take initiative (3) Know your rights (4) Raise your voice (5) Share what you’ve learned (6) Give what you want the most (7) Feel and tell the truth about what you’ve been through (8) Use it to fuel a revolution (9) Practice kindness (10) Treat your sisters’ life as if it were your own

3 The question reads: Are you aware of the existence of seven resolutions on women’s rights? *Etes-vous au courant de l’existence de sept résolutions sur les droits des femmes? Unajuwa kama kuna maazimio (makubaliano) saba* [7] *kuhusu haki ya mwanamuke?*

4 Interview with CoJ Graduate, Idwi nord, 20,160,318

5 Interview with CoJ Graduate, Uvira, 20,160,331

6 Interview with CoJ Graduate, Uvira, 20,160,331

7 Interview with CoJ Graduate Nyangezi_1 20,160,330

8 Interview with CoJ Graduate, Kamanyola, 20,160,413″

9 For example expressed in interview with CoJ Graduate Katana_1, 20,160,329

10 For example raised in Interview with CoJ Graduate, Uvira, 20,160,331,

11 Interview with CoJ Graduate, Idjwi 20,160,320. Also expressed for example by City Of Joy Graduate Goma 20,160,317

12 Interview with CoJ Graduate, Kalehe 20,160,331

13 Raised for example by CoJ Graduate Ngomo 201,603,030

14 for example expressed by a CoJ Graduate, Idjwi 20,160,320; CoJ Graduate Mboko 20,160,401

15 Interview with CoJ Graduate, Idjwi 20,160,318; also expressed in Interview with CoJ Graduate Mboko 20,160,401; Interview with CoJ Graduate Kamanyola_1 20,160,413; Interview with CoJ Graduate Idjwi 20,160,319

16 Interview with CoJ Graduate, Idjwi 20,160,318

17 Interview with CoJ Graduate Kamanyola_1 20,160,413

18 Interview with CoJ Graduate Nyangezi_1 20,160,330

19 Interview with CoJ Graduate, Kaziba 20,160,406

20 Interview with CoJ Graduate Nyangezi_2 20,160,330

21 Interview with CoJ Graduate, Lubarika 20,160,402

22 Interview with CoJ Graduate, Katana_2, 20,160,329

23 Interview with CoJ Graduate Kamanyola_2 20,160,413

24 Interview with CoJ Graduate, Kaziba 20,160,406

25 Interview with CoJ Graduate Nyangezi_1 20,160,330. Also expressed for example by CoJ Graduate Nyangezi 20,151,007

26 For example mentioned by CoJ Graduate Lubarika 20,160,402; CoJ Graduate Kamanyola_1 20,160,413

27 For example mentioned by CoJ Graduate, Goma 20,160,317; CoJ Graduate Idjwi 20,160,319.

28 Interview with CoJ Graduate, Kamanyola, 20,160,413

29 We are explicitly not considering the community perspectives in this study, as the data does not pertain to that. However, we do acknowledge that this can be an additional important element of achieving successfulness reintegration and empowerment of survivors in eastern DRC (see e.g. [11]). We do expect overall resistance to female empowerment in eastern Congolese communities, but there could also be variation in the extent to which the community appreciates (and allows) women to be more outspoken.
