# Supplementary material for: Effects of an empowerment program for survivors of sexual violence on attitudes and beliefs: evidence from the Democratic Republic of Congo
Source: Int J Equity Health. 2019 Sep 18;18:149. doi: 10.1186/s12939-019-1049-4 (PMC6751604; doi:10.1186/s12939-019-1049-4)
Supplement: Supplementary file 1 — Additional file 1: Table S1. Statistical tests for differences in empowerment on Likert scale before and after City of Joy. [file 12939_2019_1049_MOESM1_ESM.docx]

**Table S1. Statistical tests for differences in empowerment on Likert scale before and after City of Joy**

| **Statement** | **Before** | **After** | **Difference** | **p-value** |
| --- | --- | --- | --- | --- |
| Men should be community leaders | 2.8 | 2.1 | -0.7 | <0.01 |
| A women can be the boss of men in business | 3.4 | 4.3 | 0.9 | <0.01 |
| A woman can become a village leader | 3.6 | 4.5 | 0.8 | <0.01 |
| A woman can have another position of local power | 3.7 | 4.4 | 0.7 | <0.01 |
| A woman could become president of the DRC | 3.8 | 4.5 | 0.7 | <0.01 |
| It is more important for boys to go to school than girls | 1.9 | 1.6 | -0.3 | <0.01 |
| Men and women are complimentary | 4.0 | 4.5 | 0.6 | <0.01 |
| Men and women should be treated equally in church | 3.3 | 4.1 | 0.8 | <0.01 |
| Men will try to block women in the DRC from obtaining more power | 3.9 | 4.3 | 0.4 | <0.01 |
| Should have permission of husband/father before joining women's organization | 3.6 | 2.8 | -0.7 | <0.01 |
| Women have right to organize to fight for a better condition | 4.2 | 4.5 | 0.3 | <0.01 |
| A woman can be the boss of men in business | 3.8 | 4.5 | 0.7 | <0.01 |
| Men should make decisions about money | 2.7 | 1.8 | -0.9 | <0.01 |
| Men should work outside the home | 3.2 | 2.4 | -0.7 | <0.01 |
| Women should work inside the home | 2.8 | 1.9 | -0.8 | <0.01 |
| If a woman and aman do the same job, they should receive the same wage | 4.1 | 4.5 | 0.4 | <0.01 |
| Should have permission of husband/father before starting a business | 3.7 | 2.9 | -0.8 | <0.01 |
| Should have permission of husband/father before starting a new job | 3.8 | 2.9 | -0.9 | <0.01 |
| Women and men should be treated equally in business | 3.5 | 4.5 | 1.0 | <0.01 |
| Husband who beat their wives should be imprisoned | 3.8 | 4.5 | 0.7 | <0.01 |
| If a woman rejects her husband she is not a good wife | 3.0 | 2.7 | -0.3 | 0.017 |
| If wife disobeys husband, he has right to use physical means to punish her | 3.0 | 2.1 | -0.9 | <0.01 |
| Women are stronger than mean | 2.4 | 3.1 | 0.7 | <0.01 |
| Women should always do what their husband says | 3.1 | 1.9 | -1.2 | <0.01 |
| Men and women should share doing housework | 3.1 | 4.1 | 1.0 | <0.01 |
| Should have permission of husband/father before going to hospital to give birth | 3.0 | 2.3 | -0.6 | <0.01 |
| Should have permission of husband/father before going to school | 3.8 | 3.2 | -0.6 | <0.01 |
| Should have permission of husband/father before using method to avoid pregnancy | 3.8 | 3.4 | -0.4 | <0.01 |
| Women and men should be treated equally in the family | 3.3 | 4.4 | 1.2 | <0.01 |

Notes: All responses are a Likert scale, coded as follows: 5=Strongly agree; 4=Agree; 3=Neutral/Don’t know/Refused; 2=Disagree; 1=Strongly disagree. P-values were calculated using paired t-tests under the null hypothesis of no change in the answer between before and after. P-values were adjusted for multiple hypothesis testing using Benjamini & Hochberg (1995)
